# The Use of Laparoscopy Simulation to Explore Gender Differences in Resident Surgical Confidence

**DOI:** 10.1155/2017/1945801

**Published:** 2017-01-19

**Authors:** Rebecca L. Flyckt, Eliza E. White, Linnea R. Goodman, Catherine Mohr, Sanjeev Dutta, Kristine M. Zanotti

**Affiliations:** ^1^Department of Obstetrics and Gynecology, Cleveland Clinic, Cleveland, OH, USA; ^2^Stanford University Medical Center, Stanford, CA, USA; ^3^Department of Obstetrics and Gynecology, University Hospitals Case Medical Center, Cleveland, OH, USA

## Abstract

*Background.* The objective of this study was to determine whether female surgical residents underestimate their surgical abilities relative to males on a standardized test of laparoscopic skill.* Methods.* Twenty-six male and female general surgery residents and 25 female obstetrics and gynecology residents at two academic centers were asked to predict their score prior to undergoing the Fundamentals of Laparoscopic Surgery standardized skills exam. Actual and predicted score as well as delta values (predicted score minus actual score) were compared between residents. Multivariate linear regression was used to determine variables associated with predicted score, actual score, and delta scores.* Results.* There was no difference in actual score based on residency or gender. Predicted scores, however, were significantly lower in female versus male general surgery residents (25.8 ± 13.3 versus 56.0 ± 16.0; *p* < 0.01) and in female obstetrics and gynecology residents versus male general surgery residents (mean difference 20.9, 95% CI 11.6–34.8; *p* < 0.01). Male residents more accurately predicted their scores while female residents significantly underestimated their scores.* Conclusion.* Gender differences in estimating surgical ability exist that do not reflect actual differences in performance. This finding needs to be considered when structuring mentorship in surgical training programs.

## 1. Introduction

Current statistics on medical school matriculation report growing numbers of female medical students. In fact, according to records from the Association of American Medical Colleges from 2016, women now represent nearly half (49.8%) of all entering students [1]. Despite this trend, historically male-dominated fields such as general surgery (GS) continue to be predominantly male [2], whereas residencies in fields such as obstetrics and gynecology (OBG) have emerged as predominantly female.

Lack of female faculty and resident mentors during medical school, perceived gender-based discrimination, perceptions of lifestyle, and incompatibility of family life and career paths have been proposed as possible factors leading to the increased proportion of men in general surgery residencies [3–11]. Despite these factors, the number of women entering general surgery residencies increased in a linear fashion from 32% in 2000 to 40% in 2005 and the gender gap continues to narrow [12]. Concurrently, the number of female general surgery faculty mentors, albeit still low, has increased, from 12.6% in 2000 to 16.3% in 2005 [12].

Interestingly, the field of obstetrics and gynecology also includes a high volume of surgery, rigorous call schedules, and conflicts with family life, but in 2016 women comprised 80% of the OBG residency applicant pool [13]. In addition, while women now represent the majority in this surgical subspecialty, there continues to be a disproportionate number of men in the predominantly surgical post-OBG graduate fellowships of gynecologic oncology and minimally invasive gynecologic surgery.

Because both general surgery and obstetrics and gynecology encompass many of the same challenging aspects of work/life balance, it is interesting that the latter has emerged as largely female.

It is controversial whether gender differences in surgical aptitude and skill exist. Some studies have noted gender differences in abilities relevant to surgery. For example, men tend to score higher on tests of visuospatial abilities than women [14]. Despite this, a recent study of surgical simulation among surgical residents found no gender differences in laparoscopic skills [15]. These findings are similar to a 2010 prospective study of medical students, surgical residents, and attending surgeons, which showed no gender differences in coordination skills [16]. Another study of simulated laparoscopy showed that male residents completed tasks more quickly than female residents but had similar rates of errors and unnecessary movements [17]. Male medical students had more confidence about their surgical abilities than female medical students [18].

The Fundamentals of Laparoscopic Surgery (FLS) manual skills exam is a standardized, validated test of basic laparoscopic proficiency [19] that was originally developed for general surgery and recently validated for use in gynecology [20]. The goal of this study was to employ the use of the FLS exam to examine predicted and actual exam scores between men and women in the same general surgery residency as well as between men in general surgery residency and women in obstetrics and gynecology residency to determine if there is a difference in self-efficacy, or a “confidence gap,” between male and female trainees. Our hypothesis was that female residents from both general surgery and obstetrics and gynecology would underestimate their technical skills for the FLS exam compared to male general surgery residents despite no difference in ability.

## 2. Materials and Methods

### 2.1. Study Population

This study was submitted and considered exempt by the IRB at University Hospitals Case Medical Center and Stanford University. Twenty-six general surgery (GS) residents (13 male and 13 female) from Stanford University School of Medicine and 25 female obstetrics and gynecology (OBG) residents from Stanford University School of Medicine and University Hospitals/Case Medical Center were included. Three male OBG residents were excluded. GS interns were excluded from the study due to limited surgical experience at the time of test administration.

### 2.2. Data Collection Instrument and Study Design

The Fundamentals of Laparoscopic Surgery (FLS) manual skills exam is a standardized, validated test of basic laparoscopic proficiency [19]. This exam consists of five tasks: peg transfer, circle cut, endoloop, and intracorporeal and extracorporeal knot tying. A subject's technical skills are scored between 0 and 100 based on both accuracy and speed in completing the tasks. Although initially developed for nongynecologic surgeons, the manuals skills component of the FLS exam has recently also been validated in gynecology [20]. Prior to the FLS exam, residents were given a questionnaire and asked to predict their score prior to testing. The questionnaire included a statement that “an experienced laparoscopic surgeon scores approximately a 70.” They were also asked to estimate the number of simple and complex laparoscopies they had performed during residency and to indicate their year of residency, ethnicity, gender, and whether they had received prior instruction on the FLS manual skills exam. The completion times for all tasks were recorded according to standard practices. Nontimed FLS tasks were then scored by a blinded reviewer and delta values (predicted score minus actual score) were calculated for each resident. Delta values represented the correctness of a resident's self-assessment, as well as the direction of that assessment. A negative delta value indicates an underestimated score and a positive value indicates an overestimated score.

### 2.3. Data Collection and Analysis

Study subjects signed informed consent prior to participation. Data collection, review, and analysis were confidentially encoded to protect resident privacy and ensure nonbiased grading. Timed tasks could not be blinded; however all accuracy measures were scored by a blinded reviewer. Predicted scores, FLS score, and delta values were compared between residents by gender, residency type, ethnicity, year of residency, and prior instruction on FLS curriculum. Chi-square tests and independent samples *t*-test were used for univariate comparisons. A model to evaluate confounding variables affecting the predicted score, FLS score, and delta values was created with linear regression. Confounding variables were established based on those found significant with univariate analysis or those considered clinically significant. JMP version 10.0 was used for statistical analyses and a *p* value of <0.05 was considered statistically significant.

## 3. Results

There were a total of 26 GS residents (13 female, 13 male) and 25 female OBG residents that participated in the study. Three male OBG residents were excluded due to paucity of data for this group. Resident demographic and training variables are described between GS and OBG residents in Table 1. Training level was divided into the first half of training (PGY 1 and 2 for OBG and PGY 2, 3, and 4 for GS) and second half of training (PGY 3 and 4 for OBG and PGY 5, 6, and 7 for GS). Prior FLS curriculum exposure differed significantly between residents in GS and OBG. Prior FLS training was seen more frequently in GS (62% versus 20%; *p* = 0.007). Ethnicity and number of laparoscopies (both simple and complex) did not differ between groups.

Predicted FLS scores are provided in Table 2. On univariate analysis, predicted scores did not differ between residency programs (GS 40.9 ± 21.1 versus OBG 36.4 ± 17.8, *p* = 0.495), but both female GS residents and female OBG residents predicted significantly lower scores than male GS residents (Table 2). Predicted scores were lower in female GS residents than female OBG residents; however, this did not reach statistical significance (Table 2). Predicted scores differed by training level as those in the first half of training had significantly lower scores than the more senior trainees (mean difference −16.7, 95% CI −26.8–-6-6; *p* = 0.002).

Actual FLS scores are provided in Table 3. There was no significant difference in actual FLS scores between GS and OBG residencies, between female and male GS residents, between male GS residents and OBG residents, or between female GS and female OBG residents (Table 3). Scores were increased in those with prior FLS curriculum exposure (mean difference 10.6, 95% CI, 1.0–20.2; *p* = 0.031). Actual FLS scores were higher in residents in the second half of training; however this did not reach significance (53.8 ± 15.9 versus 44.9 ± 17.8; *p* = 0.069). Ethnicity had no effect on actual FLS scores (Table 3).

**Figure 1:**
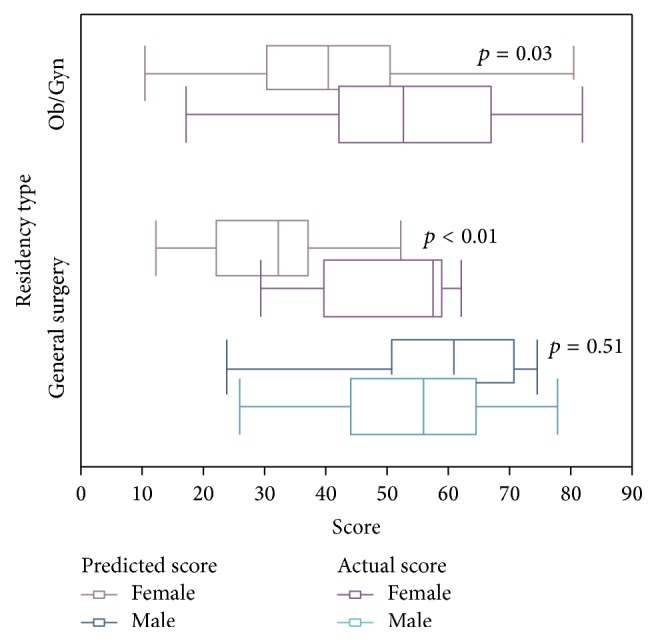
Predicted and actual scores by residency type and gender. *p* values depicted on the figure represent delta scores (difference between predicted and actual score) within the group.

Delta values (the difference between predicted score and actual score within an individual trainee, indicating the correctness of the resident's self-assessment) are depicted in Figure 1 and reported in Table 4. Delta scores did not differ between GS and OBG residencies, but there was a significant difference between delta scores by gender. Female GS residents significantly underestimated their scores by a mean of 22.5 points, whereas males overestimated their score by an average of 4.2 points (mean difference 26.7, 95% CI 12.3–41.2; *p* < 0.001, Figure 1). Results were similar when female OBG residents were compared to male GS residents, with females underestimating their scores by a mean of 11.1 points (mean difference 15.3, 95% CI 3.9–26.7; *p* = 0.010). Ethnicity, level of training, and prior FLS curriculum did not significantly affect delta scores.

Multivariate linear regression analysis was performed to identify variables influencing predicted score, actual score, and delta value (Table 5). Residency type, training level (first versus second half of training), prior FLS curriculum experience, and gender were variables included in the model. On multivariate analysis, gender and training level were significant for both predicted score and delta values. None of the variables had a significant effect on actual scores in the model (Table 5). Using ANOVA, both gender and training level had an independent significant effect on predicted score (female gender −12.5 ± 2.4, *p* < 0.01; lower level training −8.0 ± 2.15, *p* < 0.01). However, real FLS score was not affected by gender or level of training and only female gender was significantly associated with a decreased delta score (−10.1 ± 2.7; *p* < 0.01) when controlling for level of training.

## 4. Discussion

There have been a number of hypotheses for why there are fewer women seen in general surgery and surgical subspecialties. Lack of female mentorship and role models during medical school, lifestyle choice, and conflicts with family plans have been implicated in the paucity of women in surgical disciplines. This study shows that while actual scores on a validated laparoscopic teaching model do not vary between male and female trainees, females in both general surgery and obstetrics and gynecology residencies underestimated their scores versus male GS residents, despite no difference in actual scores compared to men in general surgery. This confidence gap extended across both surgical disciplines and was not reduced by the positive female surgical role models in obstetrics and gynecology. The gender differences documented here are in line with previous studies that have documented similar findings of decreased female self-efficacy [21, 22]. Lack of surgical self-confidence may contribute to gender disparity in surgical fields.

Lower feelings of self-efficacy in a female OBG residency population could explain the lower than expected percentage of female applicants in surgical OBG postresidency fellowships. A recent study from New York State demonstrated that women in OBG were significantly less likely to pursue fellowship training than their male counterparts [23]. Among respondents to this survey, 25% of male residents and 18.5% of female residents pursued postgraduate fellowship training in 2003. This disparity is especially evident in surgically oriented fellowships such as gynecologic oncology and minimally invasive gynecologic surgery. For example, 41% of applicants for minimally invasive gynecologic surgery positions in 2011-2012 were male [private communication, Fellowship in Minimally Invasive Gynecologic Surgery, FMIGs], a disproportionate finding given the preponderance of women in OBG residencies.

Higher levels of training (the third and fourth year for OBG and the fifth, sixth, and seventh for GS) were significantly associated with a higher predicted score, and those residents also had an average higher actual score, although it was not found to be independently associated with multivariate analysis. Those with a FLS curriculum did not have a higher predicted score but did have a significantly higher actual score. This may indicate that training does improve skills, but not confidence in those skills. Race did not affect predicted or actual scores, although the sample size was small.

The strengths of this study are that it was a prospective, multicenter trial with blinded grading for accuracy. It included comparison groups of male GS residents to female GS residents as well as to female OBG residents, which helps to generalize across surgical specialties. This study was limited by number of residents, specifically male OBG residents, at the specified institutions; future directions could include an increased sample size across multiple additional institutions that would encompass a broader field of residents.

Our findings have implications for resident training and indicate potential for improvement. Given that there is a difference in confidence levels in female residents that may translate to underrepresentation in fellowship and surgical practice, it is important to develop teaching styles, methods of positive feedback, and ways of increasing surgical confidence to allow for optimal education and growth in resident trainees. For example, a recent systematic review of gender differences in acquiring surgical skills concludes that “while males are more willing to practice on their own and take the associated risks, females prefer mentorship and one-on-one feedback [24].” Residents who report low feelings of self-confidence could perform additional cases with a single surgical mentor to build surgical volume and feelings of competence. In addition, it should be noted that not every female trainee in our study reported low feelings of self-efficacy. In fact, some female general surgery and obstetrics and gynecology residents reported remarkably high expected scores; characterizing the experiences and personalities of women with high levels of surgical self-confidence would be of future interest. Based on our results, training programs must be aware of potential gender differences and be prepared to individualize based on the needs and learning styles of trainees. Opportunities for mentorship as well as direct feedback from supervisors may be help to develop feelings of self-efficacy in female surgical residents.

## Figures and Tables

**Table 1:** Demographics.

Demographic	GS residents (*n* = 26)	OBG residents (*n* = 25)	*p* value
*Gender*			**<0.01**
Male	13 (50%)		
Female	13 (50%)	25 (100%)	
*Ethnicity*			0.42
Caucasian	14 (54%)	17 (68%)	
Asian	7 (27%)	5 (20%)	
African American	1 (4%)	0 (0%)	
Hispanic	0 (0%)	1 (4%)	
Other	4 (15%)	2 (8%)	
*PGY level*			0.26
First half of training	18 (69%)	13 (52%)	
Second half of training	8 (31%)	12 (48%)	
*FLS curriculum*			**0.01**
Yes	16 (62%)	5 (20%)	
No	10 (38%)	20 (80%)	
*# simple laparoscopies*			0.26
0–10	4 (15%)	7 (28%)	
10–20	6 (23%)	8 (32%)	
>20	16 (62%)	10 (40%)	
*# complex laparoscopies*			0.09
0–10	23 (89%)	17 (68%)	
10–20	3 (11%)	7 (28%)	
>20	0	1 (4%)	

**Table 2:** Predicted scores.

Group	Predicted score Mean (SD)^*∗*^	Mean difference (95% CI)	*p* value
*Residency*			
GS (*n* = 26)	40.9 (21.1)	4.5 (−6.5–15.5)	0.50
OBG (*n* = 25)	36.4 (17.8)		
*Gender*			
Male GS (*n* = 13)	56.0 (16.0)	30.2 (18.3–42.1)	**<0.01**
Female GS (*n* = 13)	25.8 (13.2)		
*Gender*			
Male GS (*n* = 13)	56.0 (16.0)	20.9 (11.6–34.8)	**<0.01**
Female OBG (*n* = 25)	32.8 (17.0)		
*Gender*			
Female GS (*n* = 13)	25.8 (13.2)	7.0 (−4.0–18.0)	0.07
Female OBG (*n* = 25)	32.8 (17.0)		
*Ethnicity*			
Caucasian (*n* = 31)	35.7 (20.2)	7.9 (−3.0–18.9)	0.15
Non-Caucasian (*n* = 20)	43.6 (18.9)		
*Training level*			
First half (*n* = 29)	31.5 (17.8)	−16.7 (−26.8–−6.6)	**<0.01**
Second half (*n* = 22)	48.2 (17.8)		
*FLS curriculum*			
Yes (*n* = 21)	42.4 (20.7)	6.3 (−4.8–17.4)	0.26
No (*n* = 30)	36.1 (18.5)		

^*∗*^Independent samples *t*-test.

**Table 3:** Actual scores.

Group	Actual score Mean (SD)^*∗*^	Mean difference (95% CI)	*p* value
*Residency*			
GS (*n* = 26)	50.0 (17.3)	2.5 (−7.4–12.4)	0.65
OBG (*n* = 25)	47.5 (17.9)		
*Gender*			
Male GS (*n* = 13)	51.8 (16.2)	3.6 (−10.6–17.8)	0.61
Female GS (*n* = 13)	48.2 (18.8)		
*Gender*			
Male GS (*n* = 13)	51.8 (16.2)	4.3 (−7.7–16.3)	0.47
Female OBG (*n* = 25)	47.5 (17.9)		
*Gender*			
Female GS (*n* = 13)	48.2 (18.8)	0.7 (−11.9–13.2)	0.91
Female OBG (*n* = 25)	47.5 (17.9)		
*Ethnicity*			
Caucasian (*n* = 31)	47.0 (18.4)	4.7 (−4.8–14.1)	0.33
Non-Caucasian (*n* = 20)	51.7 (14.7)		
*Training level*			
First half (*n* = 29)	44.9 (17.8)	−8.9 (−18.6–0.8)	**0.05**
Second half (*n* = 22)	53.8 (15.9)		
*FLS curriculum*			
Yes (*n* = 21)	55.0 (16.2)	10.6 (1.0–20.2)	**0.03**
No (*n* = 30)	44.4 (17.2)		

^*∗*^Independent samples *t*-test.

**Table 4:** Delta scores.

Group	Difference between predicted and actual score ^*∗∗*^Mean (SD)	Mean difference (95% CI)	*p* value
*Residency*			
GS (*n* = 26)	−9.1 (22.2)	2.0 (−8.9–12.9)	0.72
OBG (*n* = 25)	−11.1 (15.9)		
*Gender*			
Male GS (*n* = 13)	4.2 (17.6)	26.7 (12.3–41.2)	**<0.01**
Female GS (*n* = 13)	−22.5 (18.1)		
*Gender*			
Male GS (*n* = 13)	4.2 (17.6)	15.3 (3.9–26.7)	**0.01**
Female OBG (*n* = 25)	−11.1 (15.9)		
*Gender*			
Female GS (*n* = 13)	−22.5 (18.1)	11.4 (−23.0–0.2)	0.07
Female OBG (*n* = 25)	−11.1 (15.9)		
*Ethnicity*			
Caucasian (*n* = 31)	−11.3 (19.4)	3.2 (−7.8–14.3)	0.56
Non-Caucasian (*n* = 20)	−8.0 (20.7)		
*Training level*			
First half (*n* = 29)	−13.4 (17.3)	−7.7 (−18.1–3.1)	0.16
Second half (*n* = 22)	−5.7 (21.1)		
*FLS curriculum*			
Yes (*n* = 21)	−12.6 (21.1)	−4.3 (−15.0–6.4)	0.43
No (*n* = 30)	−8.3 (17.0)		

^*∗∗*^Negative score indicates resident underestimated real score versus what they predicted; positive score indicates overestimated real score.

**Table 5:** Multivariate analysis.

	Predicted score		Actual score		Delta value	
	Coefficient	*p* value	Coefficient	*p* value	Coefficient	*p* value
Residency type	5.4	0.09	3.1	0.40	2.4	0.51
Gender	−15.8	**<0.01**	−3.7	0.30	−12.1	**<0.01**
Training level	−6.4	**0.02**	−0.6	0.85	−5.9	**0.05**
Prior FLS training	−2.2	0.45	−6.3	0.06	4.1	0.21

## Appendix

*Resident Skills Questionnaire*

1. What year are you in residency training?
  PGY1
  PGY2
  PGY3
  PGY4
2. Have you previously received instruction in the FLS curriculum?
  Yes
  No
3. How many simple laparoscopies have you been involved in? (circle the appropriate number)
  0–10 simple laparoscopic cases (e.g. laparoscopic tubal ligation)
  10–20 simple laparoscopic cases
  >20 simple laparoscopic cases
4. How many complex laparoscopies have you been involved in? (circle the appropriate number)
  0–10 complex laparoscopic cases (e.g. laparoscopic hysterectomy)
  10–20 complex laparoscopic cases
  >20 complex laparoscopic cases
5. Predict what your FLS score would be knowing that one study [25] showed that a score at or above 70 (in a range of 0–100) corresponds to an experienced laparoscopic surgeon?
6. Gender
  Male
  Female
7. Age: —
8. Ethnicity: —
